# The role of the ceRNA network mediated by lncRNA SNHG3 in the progression of cancer

**DOI:** 10.1007/s12672-024-01184-w

**Published:** 2024-09-30

**Authors:** Ying Peng, Xi-Dai Long

**Affiliations:** 1 Department of Pathology, the First Affiliated Hospital, Guangxi Medical University, Guangxi Zhuang Autonomous Region, Nanning, 530021 People’s Republic of China; 2 Department of Pathology, Shenzhen Qianhai Shekou Free Trade Zone Hospital, Shenzhen, 518000 Guangdong People’s Republic of China; 3 Department of Pathology, the Affiliated Hospital of Youjiang Medical University for Nationalities, Guangxi Zhuang Autonomous Region, Baise, 533000 People’s Republic of China; 4 Department of Tumor Pathology, Key Laboratory of Tumor Molecular Pathology of Guangxi Higher Education Institutes, Guangxi Zhuang Autonomous Region, Baise, 533000 China

**Keywords:** Long non-coding RNA, Competitive endogenous RNAs, Tumorigenesis biomarker,, Potential therapeutic target,, LncRNA SNHG3,, Human cancer

## Abstract

**Background:**

Long non-coding RNAs (lncRNAs) are a distinct class of RNAs with longer than 200 base pairs that are not translated into proteins. Small Nucleolar RNA Host Gene 3 (SNHG3) is a lncRNA and frequently dysregulated in various human cancers.

**Objective:**

This review provides a comprehensive analysis of current research on lncRNA SNHG3, focusing on its role within the competitive endogenous RNA (ceRNA) network and its implications in cancer.

**Methods:**

A systematic literature review was conducted using PubMed up to October 2023. The search strategy included keywords such as “lncRNA SNHG3”, “competitive endogenous RNA”, “cancer”, and related terms. Studies were selected based on relevance to SNHG3's involvement in cancer pathogenesis and progression.

**Results:**

Disruptions in the ceRNA network involving lncRNA SNHG3 can impair normal cell growth and differentiation, significantly contributing to disease pathogenesis, particularly cancer. This review highlights SNHG3's substantial impact on various cancer processes and its potential as a diagnostic and therapeutic tool for aggressive cancers.

**Conclusion:**

The findings underscore SNHG3's pivotal role in cancer prevention, diagnosis, and treatment, laying a foundation for future research in cancer management. Insights from this review emphasize the necessity for further exploration and development of SNHG3-based diagnostic and therapeutic strategies.

## Introduction

The landscape of cancer epidemiology is undergoing significant transformations due to shifts in risk factors, updates in disease classification, advancements in diagnostic and therapeutic techniques, and demographic changes such as aging, population growth, and migration patterns [1–3]. Despite these advancements, cancer remains a profound global health challenge characterized by substantial morbidity and mortality [3]. While numerous signaling pathways and molecular entities have been implicated in the proliferation, invasion, and metastasis of cancer cells, the intricate molecular underpinnings of cancer pathogenesis remain incompletely understood. Therefore, further exploration into the molecular complexities of cancer, especially in the context of innovative diagnostic and therapeutic paradigms, remains paramount [4].

In genomics, non-coding RNAs (ncRNAs), particularly long noncoding RNAs (lncRNAs), have gained prominence for their regulatory roles in diverse biological processes including growth [5], development [6], and disease pathogenesis [7, 8]. Unlike messenger RNAs (mRNAs), lncRNAs, which exceed 200 nucleotides in length, do not encode proteins [9]. They play integral roles across various cellular processes from embryogenesis [10] to cancer progression [11–15], exerting broad regulatory influences. Epigenetically, lncRNAs participate in X chromosome inactivation, metabolic gene modulation, and cell cycle regulation [16], functioning at transcriptional and translational levels to activate or silence genes [17–19]. They also influence protein recruitment and RNA interactions, exhibiting context-specific functions with cytoplasmic stabilization of mRNAs and modulation of gene expression, while nuclear lncRNAs act in “cis” or “trans” roles [20]. Recent scientific literature has illuminated the intricate interplay between lncRNAs and malignancies. For instance, the association of LINC00261 with gastric cancer [21] and specific lncRNAs involved in metastasis, cooperating with TRIM16 to promote tumor invasion [22], underscore their pivotal roles in cancer initiation and progression, influencing critical processes such as proliferation, apoptosis, migration [23, 24], and chromatin remodeling [25].

The ceRNA mechanism, where lncRNAs sponge miRNAs to regulate mRNA stability and translation, has emerged as a critical regulatory pathway in cancer biology. Among the various mechanisms studied in lncRNAs, the ceRNA mechanism stands out as the most extensively researched. Small nucleolar RNA host gene 3 (SNHG3), located at 1p35.3 and predominantly found in the nucleus and mitochondria [26], emerges as a key player in cancer development, evidenced by its upregulated expression in tumors compared to normal tissues. Despite sparse comprehensive clinical research on SNHG3, its potential as a cancer biomarker remains promising. This article provides an overview of SNHG3's aberrant expression and regulatory mechanisms in tumors (Table 1), emphasizing its integral role in cancer pathogenesis through ceRNA mechanisms. Notably, recent years have witnessed a surge in SNHG3 research, particularly focusing on its ceRNA interactions with microRNAs, highlighting its pivotal role in cancer biology and suggesting novel avenues for therapeutic intervention.

**Table 1:** Clinical significance of SNHG3 in human cancer

Tumor type	Clinicopathological features	Property	Expression	Refs
Osteosarcoma	Shorter overall survival	Oncogene	Up-regulation	[35, 70]
Cholangiocarcinoma	Shorter overall survival	Oncogene	Up-regulation	[37, 71]
Prostate cancer	Advanced clinicopathological features	Oncogene	Up-regulation	[42, 68, 72–74]
Ovarian cancer	Advanced FIGO stage, lymph node metastasis poor prognosis	Oncogene	Up-regulation	[36, 38, 63, 75]
Breast cancer	Osteolytic metastasis	Oncogene	Up-regulation	[44, 48, 53, 56, 57, 64]
Non-small cell lung cancer	Shorter overall survival	Oncogene	Up-regulation	[33, 47, 51, 58, 61, 76]
Gastric cancer	Advanced stage, shorter overall survival	Oncogene	Up-regulation	[29, 30, 39, 77]
Colorectal cancer	Poor prognosis	Oncogene	Up-regulation	[50, 66, 78, 79]
Hepatocellular carcinoma	Sorafenib resistance	Oncogene	Up-regulation	[31, 40, 44, 49, 62, 80]
Laryngeal carcinoma	Migration, and invasion	Oncogene	Up-regulation	[52, 55]
Oral cancer	Migration, and invasion	Oncogene	Up-regulation	[32, 65]
Endometrial carcinoma	Tumor immune infiltration	Oncogene	Up-regulation	[67]
Bladder cancer	Shorter overall survival	Oncogene	Up-regulation	[34, 46]

## Characterization of lncRNA SNHG3 in cancers

Small nucleolar RNAs (snoRNAs) are specialized non-coding RNAs crucial for various cellular biochemical processes, primarily transcribed from protein-coding or other non-coding RNA genes. Despite lacking protein-coding ability, certain snoRNA genes contain both introns and exons, with some transcripts retaining exonic sequences to function as long non-coding RNAs (lncRNAs), termed small nucleolar RNA host genes (SNHGs) [27, 28]. One notable example is SNHG3, an oncogenic lncRNA localized in both the nucleus and cytoplasm, underscoring its multifaceted roles.

RNA-sequencing data from normal tissues sourced from the Human Protein Atlas (HPA) project [https://www.proteinatlas.org/] indicate differential expression patterns of SNHG3 across various human tissues. It shows particularly high expression levels in the bone marrow, appendix, bronchial epithelial cells, and smooth muscle cells of coronary arteries, suggesting tissue-specific roles.

**Fig. 1:**
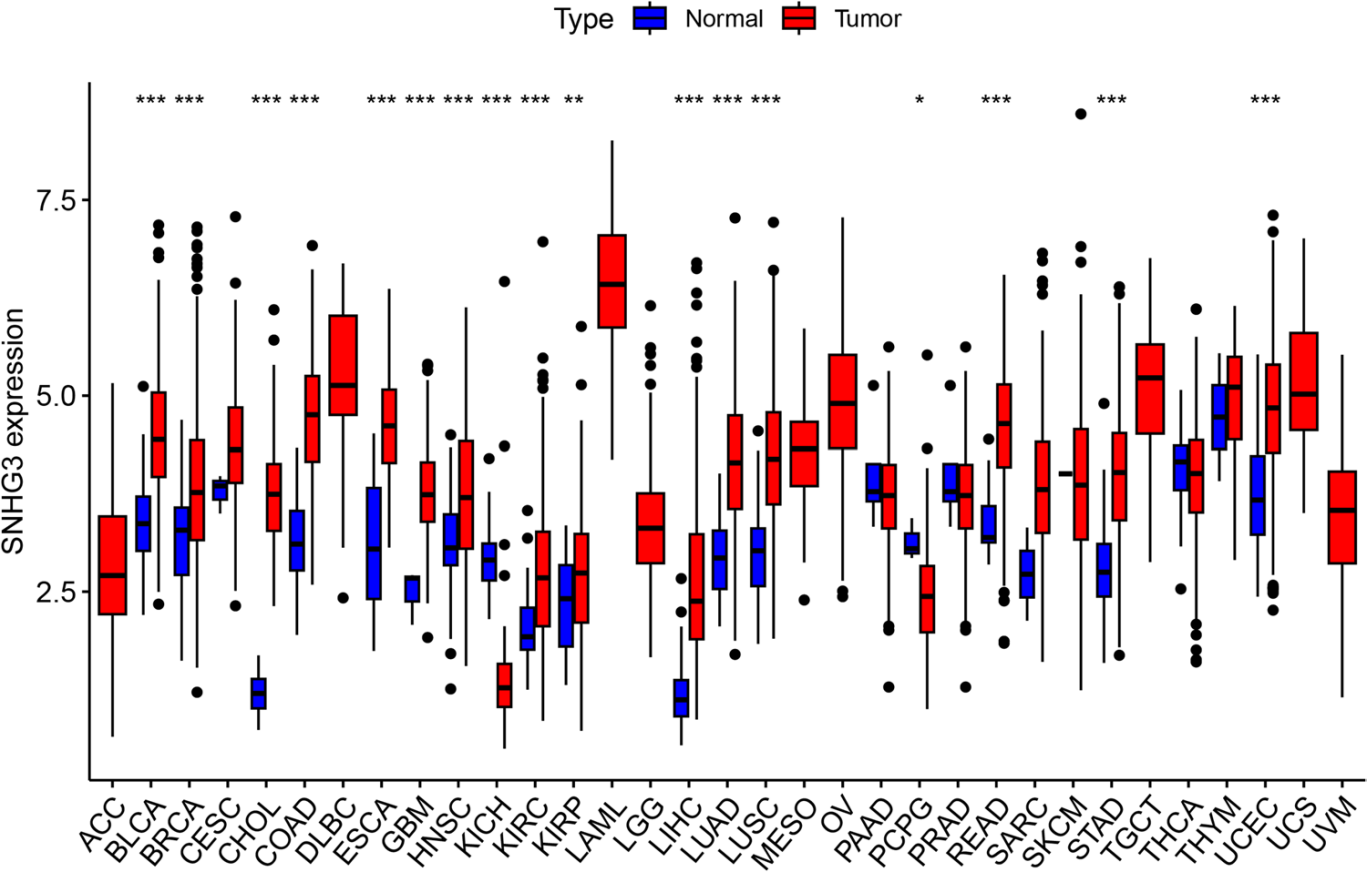
Expression Analysis of SNHG3 in 33 Types of Cancer Compared to Normal Tissues

Analysis of The Cancer Genome Atlas (TCGA) database [https://portal.gdc.cancer.gov/] reveals consistently elevated expression levels of SNHG3 across diverse tumor types compared to normal tissues. This observation aligns with findings from the LncExpDB database [https://ngdc.cncb.ac.cn/lncexpdb/]. Comparative analyses between cancerous and paired normal tissues across 33 cancer types, based on TCGA data (see Fig. 1), demonstrate significant differential expression of SNHG3 in 17 types of cancers affecting various human systems including respiratory, digestive, reproductive, urinary, and nervous systems, except where normal tissue data were limited.

The bar graph illustrates the relative expression levels of SNHG3 in cancer tissues compared to paired normal tissues across a spectrum of 33 cancer types. Data sourced from TCGA database, highlighting significant differences in SNHG3 expression across various human systems including respiratory, digestive, reproductive, urinary, and nervous systems.

In the existing literature on SNHG3, four major molecular mechanisms of action have been identified, each associated with different subcellular localizations:

### Nuclear mechanisms

A. DNA Methylation: SNHG3 plays a crucial role in DNA methylation by interacting with methylases, thereby promoting gastric cancer progression through the methylation of genes such as mediator subunit 18 (MED18) [29]. This regulatory effect is facilitated by SNHG3 binding to enhancer of zeste homolog 2 (EZH2). In a related study, Li et al.[30] investigated SNHG3's role in SEPT9 methylation and its impact on gastric cancer cell behavior, highlighting SNHG3's involvement in the complex landscape of gastric cancer development.

B. Interaction with Transcription Factors: SNHG3 interacts with various transcription factors to modulate gene expression. For instance, in hepatocellular carcinoma (HCC), SNHG3 regulates NEIL3 expression through transcription factor E2F1, implicating it as a potential diagnostic marker and therapeutic target [31]. Similarly, in oral squamous cell carcinoma (OSCC), SNHG3 influences nuclear transcription factor Y subunit gamma, promoting cell proliferation and migration and suggesting its potential as a novel biomarker [32]. In non-small-cell lung cancer (NSCLC), SNHG3 activation by E2F1 drives proliferation and migration via the TGF-β and IL-6/JAK2/STAT3 pathways, underscoring its therapeutic relevance [33]. In bladder cancer, SNHG3 regulates c-MYC to stabilize BMI1 mRNA, highlighting its role in tumorigenesis and proposing the SNHG3/c-MYC/BMI1 axis as a novel therapeutic target [34].

### Cytoplasmic mechanisms

A. MiRNA Sponging: SNHG3 acts as a ceRNA to sponge various miRNAs, forming intricate networks that influence tumor progression, including effects on cell proliferation, invasion, metastasis, and apoptosis. For example, SNHG3 upregulates Ras-related protein 22a (Rab22a) by sponging miR-151a-3p, thereby promoting migration and invasion in osteosarcoma [35].

B. Inhibition of Translation: In ovarian cancer (OC), SNHG3 regulates eukaryotic translation initiation factor 4A3 (EIF4A3) mRNA, implicating its role in energy metabolism and tumor progression [36].

A growing body of research supports SNHG3's pivotal role in cancer biology, indicating its potential as a prognostic biomarker and therapeutic target across various malignancies..

## The ceRNA network of lncRNA SNHG3

**Fig. 2:**
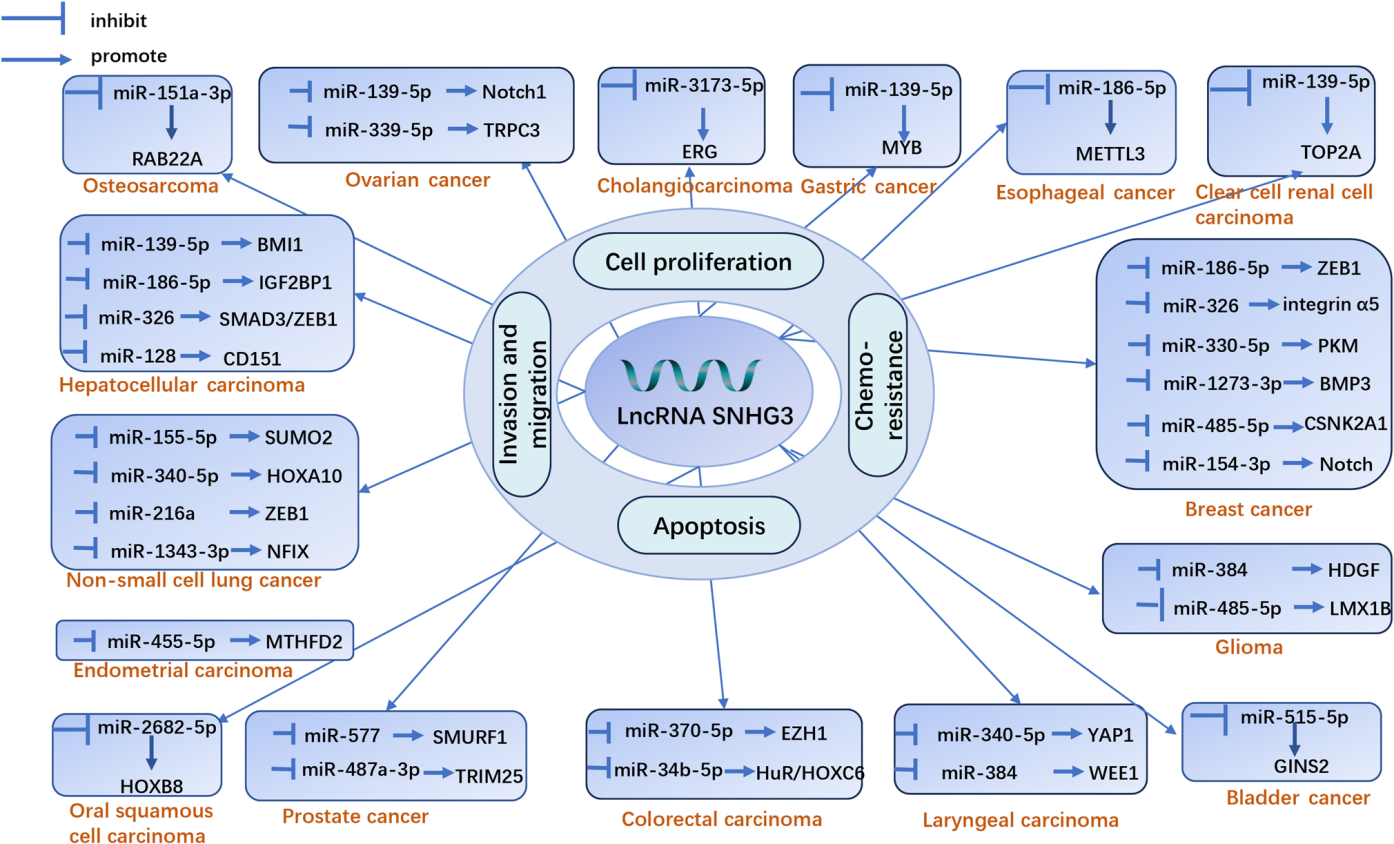
ceRNA Mechanism of lncRNA SNHG3 in Various Cancers

Long non-coding RNA SNHG3 plays a pivotal role in the pathogenesis of various cancers by modulating multiple microRNAs (miRNAs) and transcription factors. This study identifies and categorizes several regulatory axes orchestrated by SNHG3 in human cancers (Fig. 2 and Table 2).

**Table 2:** Functional roles of the lncRNA SNHG3 in human cancer

Axis	Cancer types	Cell line	Animal model	Clinical sample	Cell prolifera-tion	Invasion and migration	Cancer cell apoptosis	Chemo-resistance	Refs
SNHG3/miR-151a-3p/RAB22A	Osteosarcoma	√	–	√	–	√	–	–	[35]
SNHG3/miR-3173-5p/ERG	Cholangiocarcinoma	√	–	–	–	√	√	–	[37]
SNHG3/miR-139-5p/Notch1	Ovarian cancer	√	–	–	√	√	–	–	[38]
SNHG3/miR-139-5p/MYB	Gastric cancer	√	–	√	√	√	–	–	[39]
SNHG3/miR-139-5p/TOP2A	Clear cell renal cell carcinoma	√	—	–	√	√	–	–	[41]
SNHG3/miR-139-5p/BMI1	Hepatocellular carcinoma	√	√	√	√	√	–	–	[40]
SNHG3/miR-577/SMURF1	Prostate cancer	√	√	–	√	√	√	–	[42]
SNHG3/miR-186-5p/METTL3	Esophageal cancer	√	√	√	√	–	√	√	[43]
SNHG3/miR-186-5p/ZEB1	Breast cancer	√	√	–	√	√	–	–	[44]
SNHG3/miR-186-5p/IGF2BP1	Hepatocellular carcinoma	√	–	√	√	√	–	–	[45]
SNHG3/miR-515-5p/GINS2	Bladder cancer	√	–	√	√	√	–	–	[46]
SNHG3/miR-515-5p/SUMO2	Non-small-cell lung cancer	√	–	√	√	√	–	–	[47]
SNHG3/miR-326/integrin α5	Triple-negative breast cancer	√	–	√	√	√	√	–	[48]
SNHG3/miR-326/SMAD3/ZEB1	Hepatocellular carcinoma	√	–	√	√	√	√	–	[49]
SNHG3/miR-370-5p/EZH1	Colorectal carcinoma	√	–	√	√	√	–	–	[50]
SNHG3/miR-340-5p/HOXA10	Non-small cell lung cancer	√	√	–	√	√	–	–	[51]
SNHG3/miR-340-5p/YAP1	Laryngeal squamous cell carcinoma	√	√	√	√	–	√	–	[52]
SNHG3/miR-330-5p/PKM	Breast cancer	√	√	–	√	–	–	–	[53]
SNHG3/miR-384/HDGF	Glioma	√	–	√	√	√	√	–	[54]
SNHG3/miR-384/WEE1	Laryngeal carcinoma	√	–	√	√	√	–	–	[55]
SNHG3/miR-1273 g-3p/BMP3	Breast cancer	√	√	√	–	–	–	–	[57]
SNHG3/miR-216a/ZEB1	Non-small cell lung cancer	√	√	√	√	√	√	–	[58]
SNHG3/miR-485-5p/LMX1B	Glioma	√	√	√	√	√	–	–	[59]
SNHG3/miR-485-5p/CSNK2A1	Breast cancer	√	√	√	√	√	–	–	[60]
SNHG3/miR-1343-3p/NFIX	Non-small cell lung cancer	√	√	√	√	√	√	–	[61]
SNHG3/miR-128/CD151	Hepatocellular carcinoma	√	–	√	–	√	–	√	[62]
SNHG3/miR-339-5p/TRPC3	Ovarian cancer	√	√	√	√	√	√	–	[63]
SNHG3/miR-154-3p/Notch	Breast cancer	√	√	√	√	√	–	—	[64]
SNHG3/miR-2682-5p/HOXB8	Oral squamous cell carcinoma	√	–	√	√	√	–	—	[65]
SNHG3/miR-34b-5p/HuR/HOXC6	Colorectal carcinoma	√	√	–	√	–	–	–	[66]
SNHG3/miR-455-5p/MTHFD2	Endometrial carcinoma	√	–	–	√	√	–	–	[67]
SNHG3/miR-487a-3p/TRIM25	Prostate cancer	√	–	√	√	√	–	–	[68]

"√" indicates the presence or involvement of the corresponding factor (e.g., cell line, animal model, clinical sample) in the study of the specified axis and cancer type. "-" indicates that the corresponding factor was not applicable or not included in the study for that particular axis and cancer type

This schematic diagram details the molecular mechanisms by which SNHG3 functions as a competing endogenous RNA (ceRNA) in different cancer types. SNHG3 acts as a molecular sponge for microRNAs (miRNAs), thereby modulating multiple cancer-related signaling pathways and phenotypes such as proliferation, invasion, metastasis, drug resistance, and apoptosis.

### SNHG3/miRNA-151a-3p/RAB22A

A recent study investigated the role of the long non-coding RNA (lncRNA) SNHG3 in osteosarcoma development, employing qRT-PCR, ROC curve analysis, and Kaplan–Meier methodology. The research focused on evaluating the expression levels of SNHG3, miRNA-151a-3p, and RAB22A, alongside assessing the invasive and migratory potentials of osteosarcoma cells. The findings revealed significantly elevated expressions of SNHG3 and RAB22A, while miRNA-151a-3p levels were notably decreased. High expression of SNHG3 correlated with reduced survival rates and heightened invasive and migratory abilities in osteosarcoma cells.

Mechanistically, SNHG3 was found to sequester miRNA-151a-3p, leading to an upregulation of RAB22A expression. These results highlight the pivotal role of SNHG3 in promoting osteosarcoma progression through the SNHG3/miRNA-151a-3p/RAB22A axis. The study suggests that targeting this pathway could potentially offer new therapeutic avenues for osteosarcoma treatment [35].

### SNHG3/miR-3173-5p/ERG

In a separate study, researchers elucidated SNHG3's role in cholangiocarcinoma (CCA). SNHG3, recognized as an oncogenic long non-coding RNA (lncRNA), interacts with miR-3173-5p, a tumor-suppressive microRNA, and ERG, an oncogene. The study utilized quantitative real-time polymerase chain reaction (qRT-PCR) to assess SNHG3 and miR-3173-5p expression levels. Various assays were conducted to analyze cellular behaviors such as viability, migration, invasion, apoptosis, and protein expression in CCA. The findings revealed elevated SNHG3 expression in CCA cells, with its suppression leading to inhibited proliferation and migration. SNHG3 functions as a competitive endogenous RNA (ceRNA) for miR-3173-5p, thereby derepressing ERG and facilitating its oncogenic activities. The study concludes that targeting SNHG3 via the miR-3173-5p/ERG axis could offer promising therapeutic strategies for treating CCA [37].

### SNHG3 and miR-139-5p

Several studies have explored the relationship between the SNHG3 and miR-139-5p across various cancers. In ovarian cancer (OC), SNHG3 interacts with miR-139-5p and Notch1, promoting accelerated growth and migration [38]. In gastric cancer (GC), SNHG3 acts as an oncogene by modulating the miR-139-5p/MYB pathway, thereby driving proliferation, migration, and invasion [39]. In hepatocellular carcinoma (HCC), SNHG3 regulates proliferation and migration through the SNHG3/miR-139-5p/BMI1 axis [40]. Additionally, in clear cell renal cell carcinoma (ccRCC), SNHG3 influences proliferation and metastasis via the SNHG3/miR-139-5p/TOP2A axis [41]. Collectively, these findings underscore SNHG3's potential as a biomarker for diagnosing and treating various cancer types.

### SNHG3/miR-577/SMURF1

SNHG3 exhibits high expression in prostate cancer cell lines and promotes various oncogenic processes while inhibiting apoptosis. Research indicates that SNHG3 binds with miR-577 to regulate SMURF1 expression, which is negatively correlated with miR-577. Co-transfection with pcDNA3.1/SMURF1 was shown to reverse the effects of SNHG3 knockdown on cell proliferation, migration, epithelial-mesenchymal transition (EMT), and apoptosis. Moreover, SNHG3 has been found to promote tumorigenesis in vivo, suggesting its potential as a biomarker for diagnosing and treating prostate cancer [42].

### SNHG3 and miR-186-5p

Several studies have investigated the intricate role of specific microRNAs and lncRNAs in various cancers, highlighting the interaction between SNHG3 and miR-186. Zhang et al. [43] demonstrated that in esophageal cancer, platinum-based chemotherapy influences the SNHG3/miR-186-5p pathway, affecting m6A levels and potentially enhancing platinum efficacy. Concurrently, Wan et al. [44] revealed SNHG3's oncogenic role in breast cancer, promoting migration, invasion, and EMT through the miR-186-5p/ZEB1 axis, suggesting therapeutic avenues. Furthermore, Habashy et al. [45] explored miR-186's regulatory function in hepatocellular carcinoma, uncovering its tumor-suppressing effects by targeting oncogenic lncRNAs including SNHG3, mediated through IGF2BP1. Together, these studies provide nuanced insights into the SNHG3/miR-186 interplay across different cancers, offering potential therapeutic targets.

### SNHG3 and miR-515-5p

Studies by Dai et al. and Li et al. investigate SNHG3's role in bladder and non-small-cell lung cancer (NSCLC). Dai et al. [46] reported up-regulated SNHG3 in bladder cancer, associated with poor prognosis and promoting proliferation and metastasis via the miR-515-5p/GINS2 axis. Similarly, Li et al. [47] found elevated SNHG3 expression in NSCLC, where it inhibits cell proliferation, migration, and invasion while positively regulating SUMO2 through the miR-515-5p/SUMO2 axis. Both studies highlight SNHG3 as a potential therapeutic target in bladder and lung cancers.

### SNHG3 and miR-326

Studies by Wang et al. [48] and Zhao et al. [49] investigated SNHG3 and miR-326 in cancer. Wang et al. [48] demonstrated that SNHG3 silencing in triple-negative breast cancer (TNBC) suppresses malignancy by modulating the miR-326/integrin α5 axis and deactivating the Vav2/Rac1 pathway, suggesting therapeutic potential. Similarly, Zhao et al. [49] revealed SNHG3's oncogenic role in hepatocellular carcinoma (HCC) by acting as a competing endogenous RNA (ceRNA) for miR-326, thereby enhancing SMAD3 and ZEB1 expression. These complementary findings highlight SNHG3's diverse regulatory roles in cancer, offering new avenues for diagnosis and therapy.

### SNHG3/miR-370 -5p/EZH1

In a study investigating the role of the long non-coding RNA (lncRNA) SNHG3 in colorectal cancer (CRC) progression, SNHG3 was found upregulated and correlated with advanced tumor stages, lymph node metastasis, and poor prognosis. Employing methodologies such as qRT-PCR, CCK-8, EdU assay, and transwell assay, the research revealed that SNHG3 knockdown reduced CRC cell proliferation and invasion. This highlights the pivotal role of SNHG3 in enhancing the proliferative and invasive potentials of CRC through the SNHG3/miR-370-5p/EZH1 axis [50].

### SNHG3 and miR-340-5p

He et al. [51] elucidated SNHG3's role in accelerating non-small-cell lung cancer (NSCLC) by downregulating miR-340-5p, linking elevated SNHG3 levels with increased HOXA10 expression and decreased miR-340-5p levels, contributing to adverse NSCLC outcomes. Concurrently, Kang et al. [52] demonstrated that SNHG3 knockdown suppresses laryngeal squamous cell carcinoma progression (LSCC) by competitively binding with miR-340-5p to regulate YAP1, thereby inactivating the Wnt/β-catenin pathway. These studies underscore SNHG3's critical role in NSCLC and LSCC, highlighting its potential as a therapeutic target in both contexts.

#### SNHG3/miR-330 -5p/PKM

An article examined SNHG3's role in breast cancer growth and metabolic reprogramming. The study demonstrated that exosomes derived from cancer-associated fibroblasts (CAFs) reprogram metabolic pathways in tumor cells. SNHG3 acts as a miR-330-5p sponge, targeting PKM to control cell metabolism and proliferation [53]. These findings suggest SNHG3's involvement in breast cancer progression and its potential therapeutic implications in targeting tumor microenvironment communications.

#### SNHG3 and miR-384

Recent studies elucidated the multifaceted roles of SNHG3 and miR-384 in various cancers. Zhang et al. [54] identified SNHG3's role in glioma progression by modulating the miR-384/HDGF axis, promoting cell proliferation, migration, and invasion. Wang et al. [55] explored SNHG3's influence on laryngeal squamous cell carcinoma (LSCC) by regulating the miR-384/WEE1 axis, affecting tumor development and progression. Additionally, Ma et al. [56] highlighted SNHG3's oncogenic role in breast cancer, promoting cell proliferation and invasion via the miR-384/HDGF axis. The upregulation of SNHG3 in breast cancer underscores its potential as a therapeutic target. Collectively, these studies contribute to a comprehensive understanding of SNHG3's roles in cancer biology and its potential applications in targeted therapies.

#### SNHG3/miR-1273 g-3p/BMP3

A study investigated SNHG3's role in bone metastasis of breast cancer (BM-BCa). The study highlighted how SNHG3 modulates the miR-1273 g-3p/BMP3 axis to regulate osteogenic differentiation of bone marrow mesenchymal stem cells (BMSCs) in BM-BCa. The findings indicated that SNHG3 positively influences BMP3 expression through exosomal miR-1273 g-3p, suggesting a link between SNHG3 overexpression and osteolytic metastasis. This identifies SNHG3 knockdown as a potential therapeutic approach for BM-BCa treatment [57].

#### SNHG3/miR-216a/ZEB1

A study revealed the pivotal role of long non-coding RNA (lncRNA) SNHG3 in non-small cell lung cancer (NSCLC) [58]. SNHG3 was found significantly upregulated in NSCLC, contributing to tumor growth, migration, and invasion. Silencing SNHG3 effectively suppressed these oncogenic processes both in vitro and in vivo by modulating miR-216a and subsequently upregulating ZEB1. These findings unveil a novel pathway in NSCLC and propose SNHG3 as a potential therapeutic target.

#### SNHG3/miR-485-5p *axis*

Research demonstrated SNHG3's role in glioma by promoting tumorigenesis through miR-485-5p sequestration, leading to increased LMX1B expression and facilitating glioma cell proliferation, migration, and invasion [59]. Similarly, another study revealed SNHG3 upregulation in breast cancer, enhancing CSNK2A1 expression by absorbing miR-485-5p and recruiting the HuR protein, thereby driving disease progression [60]. These studies underscore the SNHG3/miR-485-5p axis as a common oncogenic pathway in glioma and breast cancer, suggesting SNHG3 as a potential diagnostic and therapeutic target in these malignancies.

#### SNHG3/miR‑1343‑3p/NFIX

Investigating SNHG3 in non-small cell lung cancer (NSCLC) through in vitro experiments and bioinformatics analyses, the study revealed its significant upregulation in NSCLC. It was observed to suppress cell proliferation, migration, and invasion while promoting apoptosis. Mechanistically, SNHG3 interacts with miR‑1343‑3p, downregulating its expression and enhancing NSCLC progression by increasing NFIX expression. These findings propose the SNHG3/miR‑1343‑3p/NFIX axis as a potential biomarker and therapeutic target for NSCLC [61].

#### SNHG3/miR-128/CD151

Exploring SNHG3's role in hepatocellular carcinoma (HCC), the study identified elevated expression in highly metastatic HCC cells. This elevation correlated with increased cell invasion, epithelial-mesenchymal transition (EMT), and resistance to sorafenib treatment. The oncogenic effect was mediated through the SNHG3/miR-128/CD151 pathway [62]. These findings suggest SNHG3 as a potential therapeutic target and biomarker for predicting sorafenib response in HCC, with increased SNHG3 expression correlating with poor survival outcomes and reduced treatment response.

#### SNHG3/ miR-339-5p/TRPC3

A study investigated SNHG3 in ovarian cancer (OC), focusing on its role in disease progression. SNHG3 was found to be overexpressed in OC tissues, serum, and cells, correlating with poor prognosis. Silencing SNHG3 inhibited malignant phenotypes, induced G1/G0 cell cycle arrest, and promoted apoptosis in OC cells. Furthermore, the study identified SNHG3's regulation of the miR-339-5p/TRPC3 axis, which influences OC cell behavior. These findings highlight SNHG3's potential as a diagnostic and prognostic marker in OC [63].

#### SNHG3/miR-154-3p/Notch signaling pathway

In breast cancer (BC), SNHG3's role was investigated, revealing its significant expression in BC tissues and its promotion of cell proliferation and metastasis. Knockdown of SNHG3 markedly reduced BC cell growth, invasion, and migration. Mechanistically, SNHG3 acted as a competing endogenous RNA for miR-154-3p, thereby activating the Notch signaling pathway and enhancing BC progression [64]. These results underscore SNHG3 as a promising therapeutic target in BC treatment, revealing intricate non-coding RNA interactions in cancer signaling pathways.

#### SNHG3/miR-2682-5p/HOXB8

In Oral Diseases, research elucidated molecular mechanisms in oral squamous cell carcinoma (OSCC), particularly focusing on HOXB8 regulation and its interaction with SNHG3/miR-2682-5p. Elevated HOXB8 levels were observed in OSCC tissues and cell lines, promoting cell proliferation and migration. SNHG3 was implicated in HOXB8 regulation through miR-2682-5p, validated by various experimental techniques [65]. These findings suggest potential therapeutic targets in OSCC via modulation of the SNHG3/miR-2682-5p/HOXB8 axis.

#### SNHG3/miR-34b-5p/HuR/HOXC6

A recent study investigated colorectal cancer (CRC) proliferation facilitated by extracellular vesicles (EVs) derived from cancer-associated fibroblasts (CAFs) carrying SNHG3. SNHG3, highly expressed in both CRC cells and CAFs-EVs, enhanced HuR expression by competitively binding miR-34b-5p [66]. This regulatory mechanism promoted HOXC6 transcription, thereby fostering CRC progression through the miR-34b-5p/HuR/HOXC6 axis. In vivo experiments supported these findings, suggesting promising avenues for therapeutic targeting in CRC treatment strategies.

#### SNHG3/miR-455-5p/MTHFD2

In a recent study, researchers explored MTHFD2's role in endometrial carcinoma (EC) progression. They found that MTHFD2 is upregulated in EC, correlating with poor prognosis and increased tumor immune infiltration. The study identified the SNHG3/hsa-miR-455-5p axis as a critical regulator of MTHFD2. Experimental assays confirmed that knocking down MTHFD2 inhibits malignant behaviors in EC cells, including proliferation, colony formation, and migration. Targeting MTHFD2 through the SNHG3/hsa-miR-455-5p axis could potentially serve as an effective therapeutic strategy in EC, highlighting its role as a predictive biomarker [67].

#### SNHG3/ miR-487a-3p / TRIM25

A study investigated the role of SNHG3 in prostate cancer (PCa). The research, conducted by Yu and Ren, found SNHG3 to be significantly upregulated in PCa tissues and cells. SNHG3 was observed to modulate prostate cancer cell behaviors, such as migration, invasion, and epithelial-mesenchymal transition (EMT). Mechanistically, SNHG3 acts as a sponge for miR-487a-3p, thereby regulating TRIM25 expression. Overexpression of SNHG3 promoted cell viability, migration, and invasion, while suppressing E-cadherin levels; conversely, silencing SNHG3 exerted opposite effects. This study provides valuable insights into the mechanisms of PCa metastasis and suggests potential therapeutic avenues [68].

## Significance of SNHG3's ceRNA mechanism in cancer

This section highlights the pivotal role of lncrna SNHG3's cerna mechanism in promoting cancer progression across various types, exemplified particularly in hepatocellular carcinoma (HCC).

The research by Zhang et al. [62] published in the Journal of Cellular Physiology underscores SNHG3's significant upregulation in metastatic HCC cells. Their findings reveal SNHG3's crucial functions in enhancing cell invasion, epithelial-mesenchymal transition (EMT), and resistance to sorafenib treatment. The study implicates SNHG3's modulation of the mir-128/CD151 pathway as a key mechanism, suggesting its potential as both a therapeutic target and a biomarker in HCC management.

Similarly, Yang et al. [69] identify that SNHG3 among M6A-related lncRNAs is associated with poor prognosis in patients with HCC. Their analyses highlight SNHG3's role in influencing immune infiltration patterns and conferring resistance to various therapeutic inhibitors, alongside its correlation with advanced tumor grades and stages. The study further emphasizes SNHG3's contributions to enhancing the proliferative, migratory, and invasive capabilities of HCC cells, underlining its broader implications in cancer biology.

## Conclusion

This review comprehensively elucidates SNHG3 as a pivotal regulator in cancer biology, particularly through its role as a competitive endogenous RNA (ceRNA). SNHG3 exhibits widespread dysregulation across diverse cancer types, underscoring its significance as a pivotal regulatory factor in tumorigenesis.

Our analysis highlights SNHG3's mechanism of action, whereby it competitively binds to microRNAs to modulate the expression of target genes involved in critical cancer-related processes such as proliferation, apoptosis, migration, invasion, and cell-cycle regulation. This multifaceted role positions SNHG3 as a central player in cancer progression.

Despite these insights, several limitations merit consideration. The existing literature often lacks consistency in experimental methodologies and lacks comprehensive clinical validation. Future research efforts should focus on standardizing experimental approaches and conducting large-scale clinical studies to validate SNHG3's diagnostic and therapeutic potential rigorously.

Looking forward, exploring SNHG3's interactions with other molecular pathways and identifying novel therapeutic targets within its ceRNA network present exciting avenues for future investigation. Moreover, integrating emerging technologies such as single-cell sequencing and CRISPR-based functional screens promises to deepen our understanding of SNHG3's precise role in cancer pathogenesis.

In conclusion, this review consolidates current knowledge on SNHG3's ceRNA-mediated regulatory mechanisms in cancer, emphasizing its potential as a therapeutic target and diagnostic biomarker. By addressing existing limitations and outlining future research directions, we aim to accelerate the translation of SNHG3-related discoveries into clinical applications, thereby advancing precision oncology and improving patient outcomes. A small paragraph summarizing the contents of the article, presenting the final outcome of the research or proposing further study on the subject, may be given at the end of the article under the Conclusion section.

## Data Availability

No datasets were generated or analysed during the current study.

## Abbreviations

LncRNA: Long non-coding RNA
HCC: Hepatocellular carcinoma
TNM: Tumor, Node, and Metastasis
TCGA: The Cancer Genome Atlas
ceRNA: Competitive endogenous RNAs
SNHG3: Small nucleolar RNA host gene 3
LncExpDB: Expression Database of Human Long non-coding RNAs
HPA: Human Protein Atlas
snoRNAs: Small nucleolar RNAs
SNHGs: Small nucleolar RNA host genes
MED18: Mediator subunit 18
EZH2: Enhancer of zeste homolog 2
OSCC: Oral squamous cell carcinoma
NSCLC: Non-small-cell lung cancer
Rab22a: Ras-related protein 22a
EIF4A3: Eukaryotic translation initiation factor 4A3
OC: Ovarian cancer
CCA: Cholangiocarcinoma
ccRCC: Clear cell renal cell carcinoma
TNBC: Triple-negative breast cancer
CRC: Colorectal cancer
LSCC: Laryngeal squamous cell carcinoma
CAFs: Cancer-associated fibroblasts
BM-BCa: Bone metastasis of breast cancer
HOXB8: Homeobox B8
EVs: Extracellular vesicles
EC: Endometrial carcinoma
PCa: Prostate cancer
